# Cultures and Persons: Characterizing National and Other Types of Cultural Difference Can Also Aid Our Understanding and Prediction of Individual Variability

**DOI:** 10.3389/fpsyg.2019.02689

**Published:** 2019-11-29

**Authors:** Peter Bevington Smith, Michael Harris Bond

**Affiliations:** ^1^School of Psychology, University of Sussex, Brighton, United Kingdom; ^2^Department of Management and Marketing, The Hong Kong Polytechnic University, Kowloon, Hong Kong

**Keywords:** enculturation, person and situation, levels of analysis, individualism – collectivism, eco-cultural

## Abstract

Valid understanding of the relationship between cultures and persons requires an adequate conceptualization of the many contexts within which individuals work and live. These contexts include the more distal features of the individual’s birth ecology and ethno-national group history. These features converge more proximally upon individual experience as “process” variables, through the institutional–normative constraints and affordances encountered through socialization into a diverse set of cultural groupings. This enculturation is then revealed in the individual’s response profile of values, beliefs, choices, and behaviors at any given time. Cross-cultural psychologists have typically compared these encultured responses cross-nationally by averaging the scores of equivalent groups of persons across national groups, terming these average differences “cultural differences.” This procedure has generated considerable resistance, primarily due to careless over-generalization of results to all members of a given cultural group. Critics of nation-based characterizations have challenged their methodological and conceptual inadequacies, but we now know better how to address the measurement-related aspects of culture-level “psychological” variables, such as individualism–collectivism. In challenging the accuracy of these measures, critics have also neglected to acknowledge the continuing predictive and discriminant validity of these dimensions of national culture. We here review the utility of more recent measurements. We then show how nation-level comparisons can be used by psychologists to improve our understanding of individual, rather than group, outcomes. Nations are heterogeneous amalgams of ethnicities, social classes, organizations, school systems, and families. Individuals’ socialization into these groups affects their functioning at any given point in life. These enculturations are further dependent on their gender, age, and education. Assessment of culture’s relation with individual functioning requires adequate measurement of both personality and normative aspects of situations in which behavior is enacted. Once this integration of cultural influences is achieved, the logic and methodology for integrating national culture into psychological models of individual behavior can be applied within any nation where research focuses on how within-nation cultural variation affects individual functioning. Culture, conceptualized as normative group constraints, becomes more widely amenable to study, and the hard lessons learned from cross-national research can be used to guide the practice of more locally sensitive research.

## Introduction

“Two primary goals of psychological science should be to understand what aspects of human psychology are universal and the way that context and culture produce variability. This requires that we take into account the importance of culture and context in the way that we write our papers and in the types of populations that we sample.”

-Rad et al., 2018, p. 1.

We argue in this paper that a valid understanding of the relation between persons and cultures requires an adequate conceptualization of the many different contexts within which individuals work and live. In particular, we explore the interrelation between the attributes of large cultural groups and the factors acting upon the specific experiences of individuals located within such groups. Relevant contexts include the more distal features of the individual’s birth ecology and ethno-national group history that are treated as “background” variables in Berry’s (2018) eco-cultural framework. These distal features give impetus over time toward individual experience through the constraints and affordances encountered by each individual during his or her socialization into membership of multiple cultural groupings. These extend across the lifespan from one’s family, varyingly constituted and embedded within a local community of caregivers (Keller, 2007), through schooling to various levels and associated peer group influences (Harris, 1995), to occupational settings (Lodi-Smith and Roberts, 2007), and vocational groupings (Pozzebon et al., 2010).

Over the course of this developmental process, the individual becomes less dependent on others for survival and better able to exercise greater agency in choosing his or her proximal cultural groupings and the micro-cultures of relationships within these proximal cultures (Bandura, 2001). This general process is individualized and sorted (Fowler et al., 2011) at each stage by the genetic profile of temperaments, intelligence, proclivities, and skills with which each person is endowed at birth (Thomas and Chess, 1977; Deary et al., 2010). These genetic givens are then infused into the normative cultural realities of the specific cultural groupings encountered by the individual. Successive enculturation experiences then channel the individual’s genetic profile into a behavioral repertoire which interacts with the fortuities of life (Bandura, 1998) to further refine the individual’s motivations and beliefs about the perceived world (Sasaki and Kim, 2017). These emergent structures finally channel individual choices and skill enhancements, leading to individual personality developments compatible with the constraints and affordances of the proximal cultural systems encountered as embedded within the distal birth culture of each individual.

This progressive “fitting in” across the life course may thus be construed in terms of a Bronfenbrennerian bio-ecological model of development across the lifespan (Bronfenbrenner and Ceci, 1994). This model recognizes the importance of individual genetic heritage and the epigenesis of interaction between the genetic “stuff” an individual brings into the world and the enculturation environments progressively faced by the individual throughout the life course. To understand and predict an individual’s responses at any stage in his or her life story or drama-in-the-world requires an appreciation of the cultural contexts with which he or she is interfacing.

What are these cultural contexts, and how can they best be conceptualized and measured? How are social scientists to deal with the interweaving of cultural contexts at different levels of immediacy to the living person (Bronfenbrenner and Morris, 2006)? How to incorporate the emerging polyculturalism of the individual (Morris et al., 2015a) across the lifespan into our models for behavior? What follows is our attempt to address these questions regarding culture. We first revisit conceptualizations and measurements of cultural variation. In subsequent sections we propose how such analyses of culture can be integrated with research on personality in ways that can explain the range of individual behavior more fully. Our lofty intent is to explain why an individual does what he or she does in a given cultural context at a given stage of life. Such precision of understanding and prediction is not currently available, but we hope to set an agenda for such studies.

## Cultural Variations

In developing a conceptual framework for the study of culture, psychologists have faced a series of problems. Firstly, the traditional focus of psychology has been upon understanding the behavior of individuals, and the behavior of these individuals has mostly been studied in a highly restricted set of national–cultural circumstances (Henrich et al., 2010). Consequently, it is perhaps understandable that initial attempts to understand the differences between groups of individuals from different parts of the world was often undertaken by drawing on concepts of personality (Piker, 1998). In order to move beyond such a perspective, it was necessary to take note of the way in which a given group is not only an aggregation of individuals but also a shared context moderating the manner in which a range of individual dispositions becomes expressed. A major step in this direction was accomplished by Hofstede (1980), when he proposed that variation between nations could best be summarized by defining nations as distinctive units, based on the way that they varied *from one another*, and discounting the variation between individuals within each nation. As we shall see, this procedure is not without limitations, but the recognition that in order to study culture and persons we need to differentiate levels of analysis was a critical achievement.

A second problem that has had to be faced is the question of how culture is to be defined. Everyday observation reveals a diversity of values, attitudes, beliefs, and behaviors between different groups. Each of these attributes has been included in some or all of the more than 300 definitions of culture that have been proposed (Faulkner et al., 2006). A major challenge in choosing how one can best define culture is to find a way that does not entail circularity. If variations in values or behaviors are to be considered as consequent upon cultural differences, then those same values and behaviors cannot also contribute to a definition of culture as a cause of such variations.

Two perspectives have helped toward a resolution of this conundrum. Firstly, Triandis (1995) proposed that cultural differences be examined in terms of syndromes. This concept refers to the way that cultures may be characterized not just along some single dimension, but along a series of interrelated dimensions reflecting different aspects of the social context. He differentiated five aspects of the much-discussed cultural dimension of individualism–collectivism: beliefs, attitudes, norms, roles, and values. Thus, his focus was less upon causal relationships between these attributes and more upon observing the way in which they correlated and interacted with one another at a given point in time.

A second helpful perspective is complementary to that of Triandis. Researchers have increasingly sought out variables whose measurement can contribute to the understanding of ways in which cultures evolve over time. These are the “distal” eco-social variables referred to in Berry’s (2018) model of cultural evolution, for instance measures of climate, environmental hazard, health risks, and so forth. If indices of such variables can be shown to precede contemporary expressions of cultural variations in ways that can be plausibly argued to have influenced their evolution over time, then we can begin to escape the problems of circular definition. In the following sections, we first examine the classic studies of culture-level variation, and then consider the extent to which more recent studies have overcome the criticisms that have been made of the classic studies.

### The Classic Studies of Cultural Variation

The classic studies of cultural differences have all focused upon comparisons between national cultures. As we insist, cultural differences can be studied at all levels of analysis. It is for purely pragmatic reasons that national differences have been highlighted: survey data from individuals are frequently summarized by averaging the within-nation individual responses to produce nation-level indices, and other forms of data, such as indices of wealth and inequality, are also readily available for use as tests of concurrent validity.

The three classic studies of cultural dimensions (Hofstede, 1980; Schwartz, 1994, 2009; House et al., 2004) each employed broadly similar methods. Individual-level survey items were administered to samples from a broad range of nations, with the mean score for each item then being aggregated for each nation sampled. Sample-level means were then factor analyzed, yielding a set of dimensions summarizing culture-level variation in national means. In further analyses, controls are introduced to guard against the effects of cultural variations in survey response style (Smith, 2004), and to determine the independence of the identified dimensions from variations in national wealth.

The question for consideration is what these dimension scores mean. They are based on individual-level scores, but they do not represent individuals. The scores for nations from different studies are drawn from different types of sample and have been collected at different times over the past few decades, but scores on conceptually related dimensions (for instance, individualism–collectivism and autonomy–embeddedness) are nonetheless shown to correlate substantially with one another (Smith et al., 2013). Nation-level scores have been argued to represent a collectively shared perspective on ways in which social relationships and personality are understood in a given cultural context. Thus, for instance, nations scoring high or low on power distance as a relational dynamic or on power as a value could be thought to be characterized by a particular shared understanding of the nature of hierarchy and authority (Fiske, 1991; Bond, 1996).

However, this view has become increasingly difficult to sustain. Analyses of the Schwartz database have shown that variation in values between individuals is very much greater than variation between national samples (Fischer and Schwartz, 2011), with nation-level variance for individual value-types ranging from 6 to 26%. A similar contrast between individual variance and nation-level variance is found for personality dimensions (Poortinga and van Hemert, 2001). Schwartz (2014) has addressed these results most directly, arguing that:

“Societal culture is a latent hypothetical construct. It cannot be observed directly, but can be inferred from its manifestations. The rich complex of meanings, beliefs, practices, symbols, norms and values prevalent among people in a society are among the manifestations of the underlying culture. They are not culture itself.” (p. 6).

Thus, nation-level scores can better be considered as an indicator of an underlying structure that will have been molded over time by multiple factors, and which may entail institutional structures, such as family relations, schooling, work organizations, laws, and languages spoken. They do not necessarily require value consensus. The strongest argument for retaining an interest in nation-level dimension scores is that they are found to consistently predict relevant nation-level indices derived from independent sources. The meta-analysis of relevant studies by Taras et al. (2010) found 87 significant nation-level effects for individualism–collectivism with a mean effect size of 0.46. In a similar way, Schwartz (2009) has summarized significant independent correlates of each of his dimensions. These results help to clarify what can be usefully derived from nation-level scores and what cannot. Dimension scores can give approximations of particular cultural contexts. Dimension scores do not enable predictions about the behaviors of individuals within a given sample. By adhering to this distinction, we can avoid the ecological fallacy (Robinson, 1950; Hofstede, 1980) of assuming that relations between variables at one level of analysis will be the same at a different level of analysis.

If we accept the coherence and predictive utility of some of the dimensions identified in the classic cross-national surveys, there remain numerous questions as to whether and how more recent studies can add value to what we already have. We consider first whether researchers have devised better measures and whether these have identified additional dimensions of variance.

### Improved and More Diverse Measures of Cultural Variation

The dimensions of nation-level variation that have been most fully explored are those that stem from Hofstede’s (1980) pioneering study, particularly individualism–collectivism and its associated correlate, power distance. The items used to define his dimensions were not selected on the basis of explicit theory but rather opportunistically, using an in-house morale survey conducted at IBM in the 1960s; there can be no certainty that they define the most important dimensions of variance. The classic studies have also focused principally though not exclusively on defining dimensions on the basis of items measuring personal values. Given the emphasis of Triandis (1995) on cultural variations as complex entities, there is a need to tap other syndrome components.

Using a more specifically targeted design, Owe et al. (2013) developed a measure of contextualism explicitly intended to measure the belief component of individualism–collectivism. Contextualism is defined as a belief that one can understand an individual through a knowledge of his or her context. Data from adults in 35 nations yielded contextualism scores on a six-item scale with metric invariance across national samples. Their nation-level scores were positively correlated with nation-level measures of collectivism based on values, but nation-level contextualism could also explain variance in dependent measures that was additional to that explained by values, suggesting the incremental validity of contextualism.

In a related study using the same dataset of adults from 35 nations, Vignoles et al. (2016) identified sample-level dimensions that were based on new and improved individual-level measures of self-construal. The intention of this study was to overcome the methodological weaknesses of earlier measures of self-construal and to differentiate the varying components of the global concept of collectivism. Aggregated to the sample-level, factoring of the means for self-construal items yielded seven dimensions referred to as “cultures of self,” which also achieved metric invariance. Four of these dimensions correlated significantly with earlier measures of collectivism, but the other three did not. These results underline the limitations of earlier stereotyping of Western nations as individualistic and Eastern nations as collectivistic.

Another aspect of cultural syndromes, namely the existence of cultural norms has been addressed by several projects. Some authors have tested for the prevalence of specific norms across samples. For example, Matsumoto et al. (2008) surveyed norms regulating emotional expressiveness across 32 nations. Recognizing that the relevance of norms may be context-specific, Gelfand et al. (2011) examined instead the extent to which norms in general were tightly enforced within a given cultural group. Across students in 33 nations, their six-item measure of perceived tightness–looseness was found to correlate modestly with individualism–collectivism, but to also predict aspects of cultural difference (for instance a history of social conflict) that are unrelated to collectivism. The items in the measure of Gelfand et al. (2011) emphasize perceptions of how people *should* behave, and thus provide a measure of injunctive rather than descriptive norms (Cialdini et al., 1991). A measure of the tightness–looseness of 68 nations based on descriptive norms has been created by Uz (2015). This was constructed by summarizing the variance in response to each item across a set of items drawn from the World Values Survey^1^. This index shows similar correlations with dependent measures to those obtained by Gelfand et al. (2011). Both descriptive and injunctive indices of tightness–looseness norms have potential in broadening the predictive potential of nation-level measures of cultural difference, taking the study of national culture beyond the thrall of individualism–collectivism.

The inclusion of an increasingly large number of nations within successive waves of the World Values Survey has also provided the basis for identification of further dimensions of variation. Nation-level factor analysis of selected attitude items initially identified two dimensions of variation named as rational–legal authority versus traditional authority and well-being versus survival (Inglehart and Baker, 2000). Scores on both of these dimensions were found to correlate at an average of 0.66 with Hofstede’s scores for individualism–collectivism and Schwartz’s (2009) scores for autonomy–embeddedness (Inglehart and Oyserman, 2004). Bond and Lun (2014) drew on survey items concerning the values that parents consider that their children should learn, which have been included in more recent waves of the World Values Survey. Across 55 nations, these goals were found to vary in terms of emphasis on self-directedness versus other-directedness and civility versus practicality in socializing children. The first of these dimensions was also strongly correlated with Schwartz’s (2009) dimension of autonomy–embeddedness, while the second was related to his dimension of hierarchy versus egalitarianism.

Most recently, Minkov et al. (2017) have designed survey items intended to provide a new measure of individualism–collectivism, sampling adults within 56 nations, and using a three-point response format intended to obviate variations in response style. The seven items used to define their new measure of collectivism included value statements and self-descriptions. Means were found to correlate between 0.70 and 0.89 with earlier measures. Minkov et al. (2018) have used further data from the same 56 nation survey to propose a revised conceptualization of the cultural dimension previously defined by Hofstede (2001) as long-term orientation. Minkov et al. (2018) used seven survey items to rename this dimension as monumentalism versus flexibility. Scores on this dimension of national culture correlate at no >0.32 with individualism–collectivism but correlate strongly with Hofstede’s (2001) measure of long-term orientation and with nation-level indices of school mathematics achievement. Again, national-level cultural variation extends beyond individualism–collectivism.

The more recent studies detailed in this section are notable for substantial improvements in the use of theory-driven item selection, controls for measurement error, and the sophistication of the analyses used to test for measurement equivalence. They provide some diversification away from the prior emphasis on values as the sole basis for identifying dimensions of variance. However, they continue for the most part to examine variance that is related to individualism–collectivism. Where evidence is obtained for nation-level variance along more than this single dimension, possibilities are opened up for an examination of their interactive relation to relevant dependent measures. For instance, Smith (2017) showed that across 49 nations the relationship between autonomy–embeddedness values and levels of prosocial behaviors was stronger in nations with a loose rather than a tight culture, both when using the measure of Gelfand et al. (2011) and that of Uz (2015). These types of moderation effect can be examined more thoroughly when individual-level variability is also taken into account, as we argue in detail later in this paper.

### The Search for Causal Relations

The studies outlined in the preceding section have revealed a substantial consistency in the dimensions along which nations vary, even despite variations in the measures used and the types and range of samples that have been examined. We now consider evidence of what might explain these consistencies. There are two ways in which this issue can be addressed. Firstly, if we can identify ecological circumstances that are correlated with cultural differences, but which existed prior to the emergence of these cultural differences, we shall know that it is much more plausible that the circumstances influenced the emergence of the cultural differences than vice versa (Talhelm and Oishi, 2019). Secondly, if we can detect contemporary changes in cultural differences, we can seek out circumstances that preceded those changes and are plausibly linked to them.

An example of a circumstance preceding the development of cultural differences is provided by the pathogen prevalence theory of Fincher et al. (2008) and Fincher and Thornhill (2012). These authors provided evidence that life-threatening pathogens are more frequent in some regions of the world. They reasoned that groups who developed a collective culture would be better able to survive, as they could reduce the risk of infections due to contacts with out-groups. Nations that are more collectivistic are indeed found in the hotter regions of the world where pathogens are more numerous and more dangerous.

Climate is another pre-existing circumstance, which will influence the adaptation of human populations in numerous ways, including levels of mobility and the facilitation of different types of agriculture. Talhelm et al. (2014) compared inhabitants of rice growing regions and of wheat-growing regions in China. Rice growing requires much greater hours of labor and more coordinated activity for success than does wheat growing. These authors selected samples for comparison that were closely adjacent, in order to discount regional variations. Respondents from rice-growing areas described themselves and their relations with others in more collectivistic ways.

A more complex eco-cultural theory has been advanced by Van de Vliert (2009, 2013), who reasoned that the climatic challenges posed to populations living in different regions could be moderated by the wealth that is available to contain these challenges. Levels of wealth would initially be dependent on available natural resources. Van de Vliert postulated that populations in climates with a mean annual temperature of 22°C experienced optimal “liveability.” Those living in climates that were hotter or colder than this baseline would experience increasing challenges unless these could be offset by the availability of wealth. Comparing mean scores for 15 Chinese provinces, van de Vliert et al. (2013) found collectivist responses to a survey to be correlated with level of climatic challenge. Using a similar method, involving data from more than 100 nations, Van de Vliert (2011) found three separate indicators of collectivism to be associated with climatic challenge.

Gelfand et al. (2011) were also able to provide evidence of environmental precursors across nations for the development of their dimension of tightness–looseness. They found support for their predictions that the frequencies of risks such as earthquakes, floods, food shortages, and population density during historical periods would favor the development of tight cultures. Their hypotheses were also supported on the basis of comparisons between states within the United States (Harrington and Gelfand, 2014).

Several eco-cultural explanations for the causes of cultural differences have thus been advanced. Where samples overlap to a sufficient degree, their predictions can be tested competitively against one another. For instance, Van de Vliert and Postmes (2012) showed that climate challenge predicted collectivism even after pathogen prevalence had been controlled, whereas pathogen prevalence was no longer predictive of collectivism after climatic challenge had been controlled. Thus, we can determine which are the most basic causes of cultural differences and which are more peripheral.

The second way to test for causal effects is to examine changes in scores on cultural dimensions over time. The World Values Survey has provided extensive opportunities to do so, due to repeated administrations of relevant survey items over the past several decades. A model of global cultural change was first proposed by Inglehart (1990) and has been further developed by Inglehart and Welzel (2005) and by Welzel and Inglehart (2010). We here consider the most recent formulation (Welzel, 2013). Cultural change within any particular national culture is seen as following a sequential series of stages. The first stage is cognitive mobilization, which entails an increase in availability of information and participation in educational opportunities, which can open up understandings of the ways in which culture members can utilize available resources. The experience of cognitive mobilization is predicted to lead to the development of emancipative values. These are values that favor equality, liberty, autonomy, and voice. They are measured within Welzel’s analysis by 14 value items which are factor analyzed at the individual level and then aggregated to the nation level. Where these values are experienced as fulfilled, levels of life satisfaction are predicted to become more focused on intrinsic well-being and less on material circumstances.

Welzel’s (2013) analyses showed increasing levels of emancipative values over time in almost all nations sampled. However, the rate of increase varies, with it being highest in knowledge economies and lowest in traditional economies; Li and Bond’s (2010) analysis of World Values Survey data found a similar moderation of increase in secular values by a nation’s Human Development Index (HDI) with nations at lower HDI levels showing slower increases over time. The number of nations sampled in the World Values Survey has increased greatly over time, so that for many nations data are not available for the earlier waves. By extrapolating the rates of change for a given nation for the periods where data are available to the earlier periods where it is not, Welzel was able to create scores for each nation for value change for some of the periods where data were absent. With this input accomplished, across 49 nations, he was able to test the relationship between increases in national wealth and increases in emancipative values. Controlling for wealth at Time 1, wealth at Time 2 predicts increase in emancipative values. However, controlling for values at Time 1, values at Time 2 predict increased national wealth. Thus, Welzel finds evidence for a reciprocal influence between wealth and emancipative values. Increased wealth facilitates cognitive mobilization which elicits a move to emancipative values. Enactment of emancipative values facilitates economic growth.

In a subsequent analysis, Beugelsdijk and Welzel (2018) have used World Values Survey data from 68 nations to define three dimensions of national level variance: (individualism–collectivism; duty versus joy; trust versus distrust). They judge these dimensions to be equivalent to Hofstede et al.’s (2010) dimensions of individualism–collectivism, restraint versus indulgence, and uncertainty avoidance, respectively. For these dimensions also, Beugelsdijk and Welzel (2018) found evidence for mutual influence between change in wealth and value change over time. Schwartz (2007, 2009) has conducted similar analyses, in which he shows reciprocal relations between increased national wealth and increased autonomy values, across a 10-year interval. These analyses all reinforce the conclusion that nation-level cultural values can be both a cause and a consequence of the social contexts in which they are embedded.

### How Can We Explain Nation-Level Effects?

In the preceding sections we have shown that there is some convergence in the knowledge of how the cultures of nations can usefully be described, and that there is evidence of the network of causal effects in which they may be involved. However, we have left to one side the question of just how such effects may occur. Early discussions of national culture favored defining it as a unitary state, for instance in the phrase coined by Hofstede (1980), a “collective programming of the mind.” Such definitions are no longer tenable, in light of the finding that variance in values between individuals is much greater than between nations (Fischer and Schwartz, 2011) and that there is a substantial global consensus as to which values are the most desirable (Schwartz and Bardi, 2001; Hanel et al., 2019). Furthermore, most nations comprise numerous self-evident subcultures, defined by region, religion, social class, occupation, and so forth. We need to think more clearly about the factors that may mediate nation-level effects.

Welzel (2013) proposes that while individual values denote preferences, nation-level measures indicate the relative prevalence of different values. On this view, nation-level effects are a simple averaging of individual-level effects. However, simple averaging takes no account of social inequalities within nations. Higher status individuals and groups within nations are characterized by different values from those of lower status (Kohn et al., 1990; Miyamoto et al., 2018). The greater influence of higher status groups in a nation makes it likely that their values will be better able than simple averages for the whole nation to predict nation-level effects.

We noted earlier Schwartz’s (2014) contrasting view that nation-level effects are not directly due to the prevalence of values but are due to the procedures and norms of the institutional structures embodied within nations, of which values are a reflection. An instance of such a structure that would be salient in most nations is the language used in everyday use. Kashima and Kashima (1998) found that languages used in nations that are individualistic more frequently require the use of the personal pronoun “I.” Verbs are used more frequently in collectivist nations, while nouns and adjectives are used more frequently in individualist nations (Kashima et al., 2006; Maass et al., 2006). Nouns and adjectives less frequently imply a relationship with the speaker (e.g., “He is a friendly person”), whereas verbs more often do so (e.g., “I like him”). Thus, language use may repeatedly prime individuals to think in ways that are more individualistic or more collectivistic.

A second possible way in which nation-level effects may occur is that they may be mediated by one’s day-to-day involvement in organizations, whose cultures may parallel that of the nation in which they are located. We lack sufficient studies that have concurrently sampled both national culture and organizational culture. One project in which such measures were included was the GLOBE project of House et al. (2004). The organizations from which managers provided data were within the electronics, food processing, and financial services industries. Brodbeck et al. (2004) found strong associations between national culture and organizational culture in the data from the electronics and food processing industries. However, within financial services there was stronger evidence for a global organizational culture that was unrelated to national context. Thus, there is some evidence for the view that nation-level cultural effects may be achieved by their replication within specific organizational cultures. However, in some circumstances, there is no such replication, in this case no doubt due to the international nature of the financial services sector. We also have indicative evidence that the culture of families varies in relation to national culture (Georgas et al., 2006; Kağıtçıbaşı, 2007; Keller, 2007).

### Dimensions Need to Be Understood as Dynamic Systems

In this section, we have discussed evidence relating to nation-level variability. In doing so we have retained the usage pioneered by Hofstede (1980) that refers to this variability in terms of stable value dimensions. We shall continue to do so in later sections of this paper, but in moving to the levels of analysis examined in the succeeding sections, it is important to acknowledge that we now know much more about the processes of social cognition than was the case a few decades ago. Throughout the day of an average individual, events will occur that cause a person to think of him or herself as a member of various social groups and entities, but sometimes also as an autonomous individual (e.g., Turner et al., 1987). Except in rare circumstances, they will not often think of themselves as a member of a nation. Each of these momentary identifications are likely to influence the individual’s perceptions and actions, thus creating and recreating various types of cultural influence, some of them dependent on relevant values, others more dependent on relevant norms applicable to the role they are instantiating (McAuley et al., 2002) or other features of the situation they are encountering (Reis, 2008). Recent studies have illustrated the way in which these momentary identifications can be manipulated experimentally (Hong et al., 2000; Leung and Morris, 2015; Morris et al., 2015b). Our focus here is on the way in which everyday life can prime one’s identifications in a similar manner. In this respect, the distinction between norms and values is not critical: awareness of the values endorsed by one’s peers can provide the basis for knowledge of descriptive norms (e.g., House et al., 2004; Wan et al., 2007a). Equally, the injunctive norms that characterize major institutions are likely to be reflective of the values endorsed by key groups within those institutions. National culture can be thought of as a fluid amalgam of innumerable momentary events, constantly open to change but sustained by the continuities of everyday life events in particular contexts. We seek next to tease apart some of its components.

## Reclaiming the Individual

### How Does Culture Relate to Its Members’ Individual Functioning?

As shown in the previous section, any functioning group of relating humans – dyads, families, classrooms, school districts, universities, professional associations, companies, types of organizations, ethnic communities, nations, supra-national bodies, and so forth – “has” a culture. Any group culture defines and regulates the interactions of it group members by systematizing and legitimating member exchanges to coordinate their actions for group survival and flourishing within the scope of ambient constraints and affordances. These groupings vary in their immediacy to the individual actor in daily life and may be characterized in a varying number of ways. So, for example, Lee et al. (2012) defined a family’s relationship culture using a single dimension, termed concord; Bond and Ng (2004) defined a team’s culture in terms of two dimensions of member interaction dynamics, viz., task focus and shared exchange; Hofstede et al. (1993) defined organizational practices as perceived by their members across six dimensions, like professionalism, hostility, trust, and so forth. Over the course of time, different constructs have been identified for each type of culture grouping, often without linking one set of such constructs to others previously researched in the same type of culture grouping. In consequence, the field of “culturology” is beset by a plethora of unrelated constructs.

These dimensionalizations of functioning groups constitute an unpackaging of the culture characterizing that type of grouping. So, a social scientist studying family culture may be called a “familyologist,” exploring the nomological network surrounding the cultural dimension of families being examined, as with the construct and measure of concord (Lee et al., 2012). Similarly, a social scientist may be described as a “teamologist,” an “organizationologist,” a “nationologist,” and so forth, as long as their analysis remains focused on that particular type of social grouping.

But, if social scientists wish to study individuals as individuals, how shall the researcher include the individual person’s embeddedness within an on-going cultural group in the analysis? That agenda is, after all, the fundamental role for a cross-cultural psychologist to assume. We propose here, as elsewhere (Bond, 2004, 2013) that culture may be productively treated as the *context for action* in any group setting, however large the group and however distal the cultural group’s influence on the individual. So, the personality–social psychologist could be trying to explain the behavioral response of an individual in a dyadic role relationship as defined in terms of its associated norms (McAuley et al., 2002), in an organizational setting as defined in terms of its norms of practice (Hofstede et al., 1990), or the behavioral response of an individual member of a nation as defined in terms of its norms of value or belief (see e.g., Wan et al., 2007a).

Context is thus defined as the normative structure for the group in which the individual is embedded and is acting in conjunction with other members of that group. Using these norms rather than the individual’s personal norms can yield better prediction of certain outcomes (Wan et al., 2007b). Using a person–context interaction to model behavior, we can deploy constructs and their associated measures that combine group norms in relation to the individual’s personality dispositions. A fuller understanding and prediction of individual behavior may be achieved in consequence.

### A Closer Look at Norms

Norms are statements about behavioral regularities or social expectations for desirable or proscribed behaviors, i.e., norms are either descriptive, comprising statements about what happens, or injunctive, comprising statements about what should happen (Cialdini et al., 1991; Morris et al., 2015b). Injunctive norms may refer either to behaviors that are to be done or are not to be done. The behavior in question may be concrete and specific, as in closing one’s eyes when being addressed by one’s boss, or general and wide-ranging, as in cooperation with others.

As usual in social science, norms may be specified by referring to statistical averages of the relevant constructs as reported by a sample of respondents, e.g., a value, an emotion complex, a general belief, a cognitive style, or a behavior. This average is then treated as the cultural-group descriptive norm. Alternatively, a subjective norm may be measured at the individual level by asking each individual to report his or her perception of the norm characterizing the cultural group in question. Whether and how either or both these types of norm are used will depend on the model for behavioral response being proposed by the researcher – it may be a group-level, individual-level, or cross-level model. At the group level, we have evidence that subjective norms aggregated to the nation level can account for differences in effects as well as or better than measures based on personal values (Fischer et al., 2009; Shteynberg et al., 2009). In the present case, we will discuss cross-level models where group-level norms can be related to an individual’s response across many cultural groups. In order to address this issue cross-culturally, we first examine the literature concerning personality and situation more generally.

### Situations as Normative Contexts

Given the preceding, it becomes apparent just how complex, scattered, and difficult-to-integrate is the literature on norms. The challenge is how best to include the concept of norms-as-context within a social–personality psychology that attempts to understand and predict individual responding. One approach is to acknowledge that individual responses are always situated, i.e., they apply to situations of varying specificity or generality. For instance, we may consider when a father is at home conversing with his son, or when one is in public settings with unfamiliar fellow citizens, respectively (for a cross-national example, see Matsumoto et al., 2008). Similarly, one could consider the normative constraints induced by different types of group tasks and other types of activities (Kerr, 2017). The difficulty to be addressed with this approach is that any use of norms as constructs for describing such contexts for individual responses needs to indicate both the specificity of the behaviors in question and the nature or type of situations or tasks in which each behavior is being enacted or inhibited.

Concerning situations, Lewin (1936) proposed the general formula, Behavior = *f*(Personality.Situation), i.e., B = *f*(P.S), for integrating personality factors with situational features in predicting an individual’s behavior. Norms exercise their force upon an individual’s situated behavior by suggesting or specifying the reinforcement contingencies likely to be applied should the individual behave or not behave in a normatively prescribed or proscribed way. So, some situations may be characterized as strong, others as weak depending on the intensity of reward and punishments regarded by the individual as likely to arise given certain behaviors (Kammrath et al., 2005). These reward and punishments could be applied intra-psychically or socially, alone or face-to-face (Clark et al., 2018), and be more or less effective depending on the personality of the situated individual whose response is to be understood.

The power of situations has been a longstanding assumption in social psychology based on many of the field’s dramatic early demonstrations of human responsiveness to unusual interpersonal arrangements, such as the classic Asch study on distortion of line judgments (Funder and Ozer, 1983). Using the Lewinian framework, researchers moved on to demonstrate the joint action of both personality *and* situation but made an *ad hoc* selection of specific situations to demonstrate the impact of both personality and situation in predicting individual responding (e.g., Funder and Colvin, 1991). Calls were eventually raised for a more analytic approach to distinguishing among situations as factors influencing behavior, especially in drawing a clear distinction between personality and situations as predictors in the response equation [see Reis (2008) for a historical summary].

An early taxonomy for analyzing social–interpersonal situations was provided by Mischel and Shoda (1995) with their CAPS system, which grouped situations from the perspective of the individual, based upon the individual’s cognitive–affective reactions to the situation encountered. The problem with this system was that the situation was defined by the intra-psychic responses of the individual actor and made no reference to the nature of the social situation independent of the actor’s response to that situation. A situation-referenced typology of situations independent of the individual perceiver was needed (Funder, 2009).

That gap was filled by Rauthmann et al. (2014) who developed a variety of typologies, e.g., the DIAMONDS taxonomy, which they have striven to integrate with other situation typologies in order to identify cross-study commonalities (Rauthmann and Sherman, 2018). Ziegler et al. (2019) have shown that one of these typologies, the Situation Five, shows considerable independence from a Big Five measure of personality. This enables the separation of personality measures from those of the situation, and provides evidence that the situation measures add predictive power to personality measures, supporting the original Lewinian distinction between P and S (Funder, 2006).

#### Situations as Interpersonal Interdependencies

Clark et al. (2018) have argued that relationships and the relational context within which any cognition, emotion, attitude, or behavior occurs is of fundamental importance in social–personality psychology:

“… pursuing understandings of human behavior without taking its relationship context into account runs the risk of omitting a central – if not the most central – determinant of that behavior and one that interacts in important ways with other determinants of that behavior (like personality) … of all the situational factors that might be considered in helping us to understand psychological phenomena, the relational context in which a person finds him- or herself is not only one of the most important contexts, it is probably *the* most important context.” (pp. 1–2, brackets added).

These authors proceed to make their case by examining the research data supporting the importance of variations in relationship type, relational character, and the developmental history of a relationship for their impact on a wide range of psychological outcomes – “prosocial behavior, social influence, person perception, self-concept, self-regulation, and judgments of pain, taste, beauty, and risk.” (ibid, p. 27). They conclude their review by claiming that, “… relationship contexts are one of the most powerful and pervasive situational influences fundamentally shaping human behavior.” (ibid, p. 1).

Clark et al. (2018) point out, however, that the studies in their analysis “have contrasted what occurs within close, intimate, safe, relational contexts relative to other types of relational contexts.” (p. 28). They note that other dimensions of relationship, like power differentials, are needed to supplement any analysis of relationship context. Such a broadening of relationship typology has been provided by Gerpott et al. (2018), who focused their analysis upon the interdependencies that actors perceive to vary across the breadth of various interpersonal interactions. These researchers isolated five dimensions that US and Dutch respondents use to make sense of their interpersonal interactions: “We find that people (*in situ* and *ex situ*) can reliably differentiate situations according to 5 … dimensions of interdependence: (a) mutual dependence, (b) power, (c) conflict, (d) future interdependence, and (e) information certainty.” (p. 716).

In predicting individual cooperation, Gerpott et al. (2018) showed that two of their dimensions of perceived interdependency had significant effects. The situation dimension of mutual dependence and the situation dimension of conflict increased variance explained over and above that provided by the HEXACO model of personality (Lee and Ashton, 2006) and the DIAMONDS typology for profiling situations. In future studies, other broad interpersonal outcomes, such as associative and dissociative behaviors or superordinate–subordinate behaviors, could be explored to extend this type of analysis; so, too, could more narrowly focused outcomes, like specific emotions, attributional judgments of character, or imitation of a model’s behavior. Whatever the psychological outcome of interest, using a model of perceived situational interdependency holds promise in extending research in social psychology beyond its intra-psychic, personality focus.

### Cultures as Recurring Types of Interpersonal Situations

Having identified a general line of approach, we can now explore how it could find application to the study of cultural differences. As Clark et al. (2018) claim, “Understanding how relational contexts themselves vary by culture and influence psychological phenomena is itself an important issue to address.” (p. 6). Given that cultures are systems for organizing human interaction within and between groups of interacting persons, it seems sensible to conclude that, “culture often influences individuals through the nature of their connections with others in that culture.” (ibid, p. 6). In this light, the Gerpott et al. (2018) analysis of social situations seems especially apt for comparing the norms of cultural groups concerning the management of member interdependencies. These researchers provide the following definitions of their five dimensions of interdependency:

Mutual dependence: “Degree of how much each person’s outcomes are determined by how each person behaves in that situation.”…
Power: “Degree to which an individual determines their own and others’ outcomes, while others do not influence their own outcome.”…
Conflict: “Degree to which the behavior that results in the best outcome for one individual results in the worst outcome for the other.”…
Future interdependence: “Degree to which own and others’ behavior in the present situation can affect own and others behavior and outcomes in future interactions.”…
Information certainty: “Degree to which a person knows their partner’s preferred outcomes and how each person’s actions influence each other’s outcomes.” (p. 718).

We suggest that these dimensions of social–interpersonal interdependency can be linked in suggestive ways with the dimensions of variation in national culture that we discussed in preceding sections. For instance, within collectivistic cultural contexts there are likely to be stronger perceptions of mutual dependence, less perceived personal power, and greater perceived future interdependence. Within tight cultures there is likely to be greater perceived information certainty. However, there is no reason to expect that all contexts within a given nation will replicate the characteristics of the nation as a whole. Other dimensions will become relevant, as Gerpott et al. (2018) have illustrated.

These speculations on cultural systems and their associated logics for interdependency management need to be tested empirically. They may provide a way to integrate situational analysis into cross-cultural comparisons of social and interpersonal behavior.

### The Contribution of Individual Personality

What might be the role of personality in the Lewinian model B = *f*(P.S) for explaining individual behaviors across cultural groupings? There are a few points worth considering in this regard: Firstly, whatever effect personality might exercise on the individuals involved in a cross-cultural study of behavior will be dependent upon the specific lens of the personality measure used in that study. These measures vary across a spectrum of dimensionality – from the single, as in general self-esteem (Schwarzer and Jerusalem, 1995); the double, as in proactive and prevention focus (Higgins, 1997), the triple, as in the dark and light triads (Kaufman et al., 2019); the quartet, as in McClelland’s (1987) Power, Achievement, Affiliation, and Intimacy; the Big Five (e.g., McCrae and Costa, 1989); the six of HEXACO (Ashton et al., 2014); the Great Eight Competencies (Bartram, 2005); the Implicit Motive Scale’s nine (Schönbrodt and Gerstenberg, 2012), and beyond. The choice of scale type will change depending on the model for behavioral response being used by the researcher. There is a welter of possibilities to consider.

Secondly, the personality measure used is usually completed by the respondent but could and should have been rated about the respondent by others and included in an actor–observer model of social processes (e.g., Lun and Bond, 2006). If interpersonal or social behaviors are the focus, then mutual perceptions by the actors of one another’s personalities become an element in the equation predicting the actor’s response^2^. These perceptions of the other signal to the actor holding those perceptions the likely responses by that other to the actor’s possible actions. Under certain relational considerations, perceptions of the actor held by others may be more decisive than self-perceptions in determining the actor’s outcomes in the exchange, especially over extended periods (Clark et al., 2018).

Perceptions of the actor held by the other or others can of course be considered part of the situation confronting the actor, i.e., the “real” interpersonal context. Furthermore, actors themselves differ in the accuracy of judging these perceptions held about themselves by others (Kwan et al., 2004), and perhaps more importantly, in their attentiveness to the other or the others in the equation underpinning their behavior, as research on need for closure has shown (Kruglanski and Webster, 1996). So, other-attentiveness may be considered a feature of personality, and the more objective measures of the situation mentioned above can be used in conjunction with personality to model actor behavior in group settings.

Thirdly, participants in our research endeavors enter our measurement process at different stages of their lives. By the time of a given study, their initial genetic endowment has been acted upon by the circumstances of their various socialization environments to yield the personality profile of the individual before us (Bouchard, 2004). A meta-analytic study of self-report studies of personality concluded that, “… 40% of individual differences in personality were due to genetic, while 60% are due to environmental influences.” (Vukasovic and Bratko, 2015). Environmental influences in this context would be all those factors impacting on the individual to date and not the immediate situational features considered above. Genetic information about the actor may, however, provide additional predictive power over and above the standard personality measures used in predicting behavior in free-response situations; research has shown that persons with certain genetic profiles seek out particular kinds of social situations and may be more responsive in these situations than others lacking these genetic profiles (see e.g., Dick et al., 2015; Salvatore and Dick, 2016). The extension of this work on genetic influence into the cross-cultural domain is in its infancy but promising in its capacity to add further predictive power to our standard measures of personality (Sasaki and Kim, 2017).

Finally, the Lewinian equation joining personality (P) with situation (S) to predict behavior (B) does not specify the way in which P and S, however operationalized, are to be combined – the researcher might specify and test for an additive or an interactive relationship between the two. Both approaches are possible, depending on how narrowly “the situation” is defined and whether there are “layers” of situational influence to consider in the analysis, as elaborated next.

### Culture as an Encompassing Superordinate Construct

Considering culture as a normative situational context would enable researchers to proceed in applying the Lewinian formula, B = *f*(P.S) to their behavioral response of interest. To do so, they must hypothesize the personality factors involved, the normative considerations related to the situated behavior, and any P.S interactions, and then test their model. The challenge, however, is to specify the norms applicable for the behaviors being examined. The usual procedure in cross-cultural studies is to measure an actor’s *typical* behaviors, in effect summarizing across the actor’s life to date. In that case, “the situation” in question is absorbed into the personality measure which summarizes the many, varied situations encompassing an actor’s life to date.

Such a simplifying process has been the typical approach in cross-national research. But, what if one is doing cross-ethnic, cross-state (or province), cross-organizational, cross-team, or cross-family research across national cultures where variations of ethnicity, provinces or states, organizations, teams, and families are embedded within nations? In this case, cultural influences on social psychological processes would “seep down” to the situational–relational level through many potential layers of culture, varying in their immediacy to the actor. A multi-layered, multi-level model of influence then becomes necessary to disentangle the complexity of possible cultural influences in play.

### How Does Culture Exercise Its Effect on Individual Social–Psychological Outcomes?

Our approach outlined above is consistent with Brady et al.’s (2018) assertion that, “Fully understanding human behavior necessitates understanding the cultural influences *on individuals in a given context*.” (p. 11,407, italics added). If researchers are to use the Lewinian formula for behavior as a guide, then behavior, B, can be the result of variation in individuals’ personality, P, variation in the immediate normative situation, S, and variation in their interaction, (P.S). When researching across cultures, culture then becomes a superordinate construct in which the B = *f*(P.S) model is embedded, a higher-level summary of the individual’s situations encountered across a lifetime.

A number of possible effects may emerge from this multi-cultural, cross-level analysis:

(1) P may exercise a main effect across all cultural groups, positioning the “average behavior” of members from any cultural group differently from that of other cultural groups.
(2) S may exercise a main effect across all cultural groups, positioning that average behavior of members from any cultural group differently from that of other cultural groups.
(3) The P.S interaction may exercise a main effect across all cultural groups, positioning that average behavior of members from any cultural group differently from that of other cultural groups. Leung and Bond (1989) refer to these three possible effects as “cultural positioning effects,” where culture is the superordinate context for action and S the immediate situation confronting the individual actor.

These types of analysis require multi-level modeling (Raudenbush and Bryk, 2002; Nezlek, 2011). We have emphasized throughout this paper the importance of distinguishing between levels of analysis, and of not assuming that the relations between variables are the same at different levels of analysis. Multi-level modeling provides the best currently available procedure for detecting these probable differences in relationships. To be effective, an adequate number of samples is required to estimate the relations between variables at each level of analysis. This requirement is typically most challenging for higher-order samples such as nations. While multi-level models containing no more than 10 higher order samples may be tested, the likelihood that hypotheses can be validly tested is greatly enhanced where 30 or more samples have been included.

As an example of this approach, consider the work of Becker et al. (2012). These authors asked adolescents in 21 nations to provide 10 answers to the question, “Who are you”? Respondents were then asked to rate each attribute of themselves on a series of dimensions, including how much the attribute provided distinctiveness and how much it was important to defining who they were. Individual-level analysis showed that distinctiveness was important in all samples. However, multi-level modeling showed that in more individualistic samples distinctiveness was associated with seeing oneself as separate and different, whereas in collectivistic samples distinctiveness was associated with membership of distinctive groups. Thus, there were main effects both for persons (P), for cultural contexts (S), but Becker et al. (2012) also found significant P.S interactions.

This study lacked individual-level measures of S, but where these are also included we may anticipate that a variety of cross-level moderation effects will emerge. So, culture, however operationalized, might interact with P, with S, with P.S, or with any combination of factors to influence individual behavior, B. These cross-level moderations are referred to by Bond and van de Vijver (2011) as “cultural salience effects,” such that the cultural background of an individual results in that individual’s position on P, S, or P.S being relatively more or less powerful in predicting his or her behavior than for an individual from another culture.

“Cultural salience effect” is a descriptive phrase only, requiring a persuasive logic for explaining why this way of unpackaging culture results in greater or lesser weight being attached to P, to S, or to P.S in affecting B. An *a priori* argument, well grounded in the literature involving this type of cultural grouping – nation, organization, team, family, role dyad – would be needed to provide a persuasive argument for conducting such a study. As always, methodological rigor and statistical appropriateness are required to prove one’s case. Since results do not always support the researcher’s hypotheses, so a sensible, fair-minded interpretation of the findings would provide a necessary conclusion to the research exercise (Bond, 2019).

When designed using the Lewinian formula described above, cross-cultural research can improve psychologists’ interpretive power. “When psychologists leverage interpretive power, they can use cultural differences to build theories that explain a greater range of phenomena with greater nuance.” (Brady et al., 2018, p. 11,408). Carefully planned, executed, analyzed, and interpreted cross-cultural studies will enable researchers to “… go beyond simply documenting cross-cultural differences; (instead) they use their understanding of how culture shapes cognition, motivation, and emotion to build theories that explain why, how, and when psychological processes manifest differently in diverse cultural contexts.” (ibid., p. 11,407, brackets added). That increase in precision and generalizability should be our goal as *cross-cultural* psychologists.

## Into the Future

We have argued that the culture of any type of group may be defined as the norms characterizing that type of group in situated interaction with the personalities of its members to yield each member’s response. This conceptualization of the cross-level relationship between culture and the psychology of its members opens up a host of possibilities for modeling individual behavior. Our vision has been one of a possible future. Few studies exist that have analyzed effects that included individual-level variance in terms of social context other than that defined by nation-level dimensions. We conclude by identifying three studies that indicate the variance that remains to be explored through more detailed investigation.

Diener and Diener (1995) examined the correlation between self-esteem and life satisfaction among students in each of 31 nations. The strength of the correlations was uniformly positive but varied in magnitude. Diener and Diener (1995) found that the strength of the correlation within each sample was significantly predicted by indices of individualism–collectivism. However, the measures of individualism–collectivism were not provided by the student respondents. Direct measurements would give a stronger test of their conclusions. Similar results were reported by Schmitt and Allik (2005) who sampled students from 53 nations. Self-esteem was found to correlate positively with extraversion and negatively with neuroticism in almost all samples, but the magnitude of the relationships varied, and this magnitude was in some instances predicted by scores on Hofstede’s cultural dimensions.

A more recent study did involve direct sample-level measurement of cultural differences. Lun and Bond (2016) examined the strength of the linkage between trust of one’s ingroup members and satisfaction with life in 65,021 representatively sampled individuals from 50 nations. These nations were conceptualized as differing from one another along two dimensions of socialization goals for children, viz., Self- versus Other-directedness and Civility versus Practicality. Although these dimensions are derived from individuals’ recorded preferences, they were identified through sample-level factor analyses that discount individual-level variance, and are thus true estimates of sample-level variability. These dimensions would be considered as process features of culture in Berry’s (2018) eco-cultural model of ecological features of the nation. Using Berry’s terms, these measures of national socialization also relate to “psychological” features of each nation, for instance the norm for equality that characterizes its citizens (Bond and Lun, 2014).

Moving from the national level of analysis to the individual level, Lun and Bond (2016) found that trust of ingroup members was a predictor of life satisfaction pan-nationally but was a *stronger* predictor in nations higher in Self-directedness and in Civility. The socialization context for children characterizing one’s national culture thus has relevance to the life satisfaction of individual citizens. However, because this study also reported results nation by nation, they were able to show that trusting one’s ingroup did not predict life satisfaction significantly in *all* 50 nations, even if those nations were high in either Self-directedness or Civility. In fact, for Ethiopians the relationship was negative, albeit not significantly so. Why?

Of course, the specific result for Ethiopia may be an anomaly due to measurement error. However, to understand the results obtained more fully, we should need to move closer to the type of research design outlined in the preceding section. Rather than using a measure of generalized trust as the predictor, we should require measures of personality dimensions and measures of the perceived attributes of different interaction contexts. The World Values Survey already includes a very brief measure of personality but measures more akin to those proposed by Gerpott et al. (2018) would also be required.

Here, then are opportunities for a more “nuanced” understanding of the social–psychological phenomenon in question, albeit one that raises provocative questions of generalizability, begging the question of why a variable that predicts life satisfaction in most national cultures does not do so in a few. Such provocation is surely a reason for conducting studies as culturally comprehensive as that of Lun and Bond (2016), despite its relatively limited focus on culture just as national context. After all, each individual in these 50 nations is embedded within a family of origin (or family of creation), which is itself embedded within a linguistic–ethnic cultural group within a provincial or state cultural group. Each of these levels of culture may exercise further moderating effects upon the strength of linkage between ingroup trust and life satisfaction. Assessing these further “nuancings” of the outcome requires careful theorizing to justify their examination, not to mention considerable statistical sophistication in their application. But, is not such nuancing in the interests of generalizability the purpose of doing psychology cross-culturally? We agree with Brady et al. (2018) in asserting that it is.

“Everything should be made as simple as possible, but no simpler.”-Albert Einstein

## Author Contributions

Both authors listed have made a substantial, direct and intellectual contribution to the work, and approved it for publication.

## Conflict of Interest

The authors declare that the research was conducted in the absence of any commercial or financial relationships that could be construed as a potential conflict of interest.

## Footnotes

1 www.worldvaluessurvey.org

2 http://www.persoc.net/
